# The Pathology of Acute Rheumatism

**Published:** 1903-09-12

**Authors:** 


### THE PATHOLOGY OF ACUTE RHEUMATISM.

In a recent issue of *Guy's Hospital Gazette* Dr. Ainley Walker points out that a mass of evidence has been accumulated in the past six years which leads to the conclusion that a special micro-organism is constantly associated with rheumatic fever, and can be isolated from the lesions which occur in the course of the illness. This organism can be cultivated through successive generations in the laboratory, and upon injection into animals has caused pyrexia, aneurism, acute arthritis, endocarditis, pericarditis, myocarditis and iritis. The tendency has been for these lesions to recover completely, and repeated injections have caused successive attacks of arthritis. It is to be noted that severe infections have terminated in fungating endocarditis associated with visceral infarctions. The organism, according to Dr. Ainley Walker, varies in structure and appearance according to the character and composition of the culture medium employed, hence it has been described by different observers as a diplococcus, a streptococcus, and a strepto-diplococcus. Probably in an ordinary routine examination it would be reported as a streptococcus. However, Dr. Walker considers the name of micrococcus rheumaticus is applicable to the organism. It may be distinguished from ordinary streptococci by certain cultivation tests, among which may be mentioned the faculty of growing in filtered cultures of ordinary streptococci, and the rapid production of considerable quantities of acid. For some sixteen months Dr. Walker and Mr. Ryffel have been investigating the production of acid which is such a marked feature of rheumatic fever and of the laboratory cultivation of the micrococcus rheumaticus. About a year ago they were able to state that the acid in question was not lactic acid, and they now put forward the following propositions:—(1.) The micrococcus rheumaticus produces formic acid in very considerable quantity, and also at least one other acid of the fatty series. (2.) Formic acid is present, not only in the filtered cultures, but also in the bodies of the micrococci themselves. The washed micro-organisms contain also at least one of the higher fatty acids. (3.) Ordinary streptococci only give rise, so far as they have as yet been able to determine, to a small quantity of formic acid in similar media. This observation promises to constitute a further distinction between the micrococcus rheumaticus and other members of the streptococcus group. (4.) In acute rheumatism formic acid appears in the urine in most appreciable quantities. In normal urine it is only present in minute amounts. (5.) Under the salicylic acid treatment of rheumatic fever, the amount of formic acid in the patient's urine is diminished.

